# Phospho-tau 181 is enhanced in saliva and plasma of edentulous patients: a first sign of dementia?

**DOI:** 10.3389/froh.2025.1627681

**Published:** 2025-07-30

**Authors:** Christine Zürcher, Michaela Defrancesco, Christian Humpel

**Affiliations:** ^1^ University Hospital for Conservative Dentistry and Periodontology, Medical University of Innsbruck, Innsbruck, Austria; ^2^ Department of Psychiatry, Psychotherapy, Psychosomatics and Medical Psychology, Division of Psychiatry I, Medical University of Innsbruck, Innsbruck, Austria; ^3^ Laboratory of Psychiatry and Experimental Alzheimer’s Research, Department Psychiatrie I, Medical University of Innsbruck, Innsbruck, Austria

**Keywords:** saliva, phospho-tau181, Alzheimer’s disease, edentulous, periodontitis, biomarker, diagnosis

## Abstract

**Objectives:**

There is evidence that periodontitis can enhance the progression of Alzheimer's disease (AD). The biomarkers beta-amyloid (40 and 42), total tau, and phospho-tau181 (pTau181) in cerebrospinal fluid help to diagnose AD. Saliva is an easy-to-collect fluid and we aim to analyze these biomarkers in patients with periodontitis.

**Subjects and methods:**

Four groups of individuals were included: patients with healthy periodont, those with mild and severe periodontal diseases, and edentulous patients. These four biomarkers were analyzed in saliva using Lumipulse technology, and as a control, also cortisol, transferrin, and interleukin-6 were measured. Patients underwent a neuropsychological assessment, and plasma pTau181 was analyzed.

**Results:**

No changes were seen for salivary beta-amyloid and total tau; however, salivary pTau181 was significantly increased in edentulous patients. This was accompanied by enhanced plasma pTau181 levels.

**Conclusion:**

Our data show that pTau181 was significantly higher in saliva and plasma of edentulous patients, and we suggest that the loss of teeth may be linked to the progression of dementia. Consultation of dentists may be of importance to find risk factors for the progression of dementia, and salivary pTau181 could be a new associative marker.

## 1 Introduction

The life expectancy of humans has increased in the last 100 years. As age is the main risk factor for Alzheimer’s disease (AD), the number of patients suffering from AD will dramatically increase in the coming years, to the point that about 152 million dementia patients can be expected—worldwide—by 2050 (1). AD is characterized by severe β-amyloid (Aβ) deposition in the brain (extracellular plaques), tau pathology (hyperphosphorylated tau causes neurofibrillary tangles), cell death of cholinergic neurons (loss of the neurotransmitter acetylcholine), astroglial and microglial activation, inflammation, and cerebrovascular damage (2). The reasons for people developing AD are not known. The Aβ cascade hypothesis claims that a mutation of amyloid-precursor protein (APP) causes the disease, followed by tau pathology (3). However, this hypothesis is now surrounded by more and more controversy (4, 5). Alternatively, others suggest that tau may also induce AD, as tau pathology accounts for frontotemporal dementia without any Aβ pathology (6, 7). Another hypothesis suggests that inflammation or damage of the blood–brain barrier (BBB) may contribute to the disease (8, 9).

The definitive diagnosis of AD requires both a clinical diagnosis of the disease and a postmortem detection of AD pathologies. A probable diagnosis of AD can be established—with a confidence of >90%—based on clinical criteria, such as medical history, physical examination, laboratory tests, neuroimaging, and neuropsychological evaluation. A promising area of research for the laboratory diagnosis of AD is the analysis of cerebrospinal fluid (CSF), where the measurement of Aβ(40) and Aβ(42), total tau, and phospho-tau-181 (pTau181) can distinguish AD patients from healthy subjects with high specificity and sensitivity (10–12). Unfortunately, the use of CSF biomarkers is limited by its invasive collection. Thus, there is a need to discover biomarkers in other human biological fluids—such as blood, urine, or saliva—which permit for the collection of a high number of samples in a cost-effective way.

Saliva is a complex and heterogeneous human fluid that is easily accessible, without any ethical concerns, but is still unrecognized as a source of biomarkers for AD. Whole saliva is composed of water, peptides and proteins (including hormones and enzymes), sugars, lipids, and electrolytes and contains huge amounts of oral bacteria. The saliva metabolome appears to be comparable to the human serum and CSF metabolomes, in terms of chemical complexity and the number of compounds (13). The saliva proteome contains approximately 2,300 proteins, 27% of which are identical to plasma proteins (14). This is consistent with the data from previous studies, which showed that compounds found in human saliva are usually found in human blood, although at different concentrations (15). Thus, saliva is a powerful and accessible human fluid and a future tool for AD diagnosis (16–20) and also well discovered in our laboratory (21, 22). We recently reviewed the use of saliva for diagnostic purposes, especially for AD (23). It was already shown that AD biomarkers can be detected and measured in saliva samples (22).

The identification of periodontitis as a significant risk factor for developing AD has already been postulated in 1992 (24), which seems to be well established now (25, 26–33, 75). The idea that the oral microbiome may link periodontitis and AD has been hypothesized (34, 35). In fact, an infection with *Porphyromonas gingivalis*—an asaccharolytic, gram-negative, anaerobic bacterium—plays a key role in the development of periodontitis and further has been considered a risk factor for the progression of AD (36–42). In addition, it was shown that oral pathogens or their metabolites can directly invade the brain and cause neuroinflammation (34, 43, 44). Further, oral pathogens can induce gut dysbiosis, which changes intestinal permeability, resulting in inflammation or damage of the BBB and allowing more pathogens to enter the brain (45–50). Furthermore, periodontitis results in systemic low-grade chronic inflammation (51) and can induce neuroinflammation and influence brain function (34, 43, 44).

The enormously high numbers of presumed AD patients call for further establishment of reliable diagnostic or associative markers. Thus, the aim of the present study was to investigate AD biomarkers in the saliva of elderly people with different periodontal conditions (including edentulous patients) and further examine their cognition, to obtain a better understanding for the interaction of different clinical findings and laboratory results, and to establish a valid and non-invasive diagnostic tool for the diagnosis and monitoring of both disease and possible progression to AD.

## 2 Material and methods

### 2.1 Participants in this study

In this study, 82 volunteers were recruited in the Department of Dental and Oral Medicine and Cranio-maxillofacial and Oral Surgery of the Medical University of Innsbruck (Austria) between 4 May 2022 and 23 June 2024. All subjects (older than 60 years) were allocated to one of four groups: group 1 with edentulous patients who lost all teeth at least 6 months ago. Probands in group 2–4 had at least a minimum of 10 teeth and were assigned as follows: group 2 without advanced periodontal disease (52) (community periodontal index of treatment needs, CPITN score 1 or 2), group 3 with mild/moderate periodontitis (CPITN score 3), and group 4 with severe periodontitis (CPITN score 4). Inclusion criteria were age ≥60 years, male or female, contractual capability, and ability to follow instructions. Exclusion criteria were missing consent, decompensated metabolic disease, acute myocardial infarction, acute cancer, current or previous alcohol abuse and dependence, major neurological or psychiatric disease (e.g., stroke, epilepsy, schizophrenia), infectious diseases (e.g., HIV, hepatitis B, or COVID-19), acute parotitis, any form of spontaneous bleeding in the mouth, edentulous subjects with dental implants, patients post radiation in the head/neck area, xerostomia (unstimulated salivary flow ≤200 µL per 2 min), and severe cognitive impairment.

At the beginning of the study, the probands were informed about the study procedure, and the inclusion and exclusion criteria were proved. After informed consent was obtained from them, they were asked to answer several questions regarding their tea, coffee, and alcohol consumption, their nutritional habits, their smoking status, any surgeries or diseases (e.g., diabetes), and reasons for former tooth loss. The Ethics Committee of the Medical University of Innsbruck, Austria, approved the study (ID EK 1270/2022). The study was conducted in accordance with the 1964 Helsinki Declaration and its later amendments. All subjects signed an informed written consent form prior to the study enrollment.

Clinical oral examination was conducted after saliva samples were taken. All remaining teeth (>10) were examined at four points (mb, db, ml, and dl) using a periodontal probe (Parodontometer PCP12, Hu-Friedy Mfg. Co., LLC, Chicago, IL, USA). The CPITN was examined, with the following scale: CPITN 1 or 2: bleeding on probing or calculus; CPITN 3: pocket probing depth up to 5 mm; CPITN 4: pocket probing depth 6 mm or more. Thus, the probands could be allocated to one of the four groups. All edentulous patients were wearing complete dentures. During the clinical examination, the oral mucosa was evaluated and a panoramic radiograph was performed. In addition, the hygiene status of the dentures was assessed using a visual index.

*Collection of saliva* was done immediately before oral examination. All subjects were asked to refrain from eating, drinking, smoking, and practicing oral hygiene 2 h prior to saliva collection. The probands had to spit for exactly 2 min into a 50 mL preweight Falcon tube (unstimulated whole saliva), which was immediately sent to the laboratory for analysis.

#### 2.1.1 Collection of blood samples

The blood samples were taken after the neuropsychological assessment (in a 10 mL EDTA tube) and were sent immediately to the laboratory for analysis.

### 2.2 Neuropsychological assessment

All patients were invited for a neuropsychological assessment at the regional memory clinic within 6 months after dental treatment. Of the 82 patients invited, 39 were willing to complete a neuropsychological assessment, which took about 40 min. Patients were tested on verbal memory (word list learning, word list delayed recall, and word list recognition), figural memory [free recall, Consortium to Establish a Registry for Alzheimer's Disease (CERAD)], object naming [Boston Naming Test (BNT)—short version, CERAD], categorical verbal fluency (animals/min, s-words/min, CERAD), and psychomotor speed/mental flexibility (Trail Making Test A and B) of the CERAD battery (53). In addition, the Mini Mental State Examination (MMSE) (54) and clock drawing test (CLOX) test (55) was administered. Depressive symptoms were assessed using the 30-item Geriatric Depression Scale (GDS) version (56).

#### 2.2.1 Processing of saliva

All samples were processed within 3 h. The weight of the tubes with saliva was measured (for calculation of salivary flow), then centrifuged at 3,000 × *g* for 5 min, the supernatant collected, and the volume measured. The four Alzheimer biomarkers were analyzed immediately (1 + 1 diluted) and the rest frozen at minus 80°C.

### 2.3 Analysis of biomarkers

Levels of *salivary Aβ(40), Aβ(42), total tau, and pTau181* were measured using automated Lumipulse enzymatic light emitting technology (Fujirebio G600II) as reported by us (22). The Lumipulse assay is an automated roboter platform, using an enzymatic light emitting system. This system gives very fast and accurate values within 35 min. The single racks are placed in the system and each unit contains a triple tube with antibodies and magnetic beads (see https://www.fujirebio.com/en/products-solutions/lumipulse-g600ii).

*Total protein* was determined in undiluted saliva using the Bradford assay as described by us and the AD biomarkers corrected to 1 mg/mL total protein (22).

*Cortisol levels* were analyzed by using a commercial Enzyme Immune-ELISA (Salimetrics, 1–3102, USA). Briefly, 25 µL saliva (diluted to 1 mg/mL) was added with 200 µL conjugate to the well, incubated 1 h at room temperature (RT), washed, developed with substrate, and measured at 450 nm. The detection limit was 0.037 µg/dL.

*Transferrin* levels were analyzed by using a commercial Enzyme Immune-ELISA (Salimetrics, 1–1302, USA). Briefly, 20 µL saliva (diluted to 1 mg/mL) was added with 50 µL conjugate and 50 µL antiserum to the well, incubated 45 min at RT, washed, developed with substrate, and measured at 450 nm. The detection limit was 0.4 mg/dL.

*Interleukin-6* (IL-6) levels were analyzed by using a commercial Enzyme Immune-ELISA (Salimetrics, 1–3602, USA). Briefly, 100 µL saliva (diluted to 1 mg/mL) was added to the well, incubated 1 h at RT, washed, then 100 µL conjugate was added, incubated 2 h at RT, again washed and 100 µL of the streptavidin-HRP conjugate added, incubated 20 min at RT, washed and developed with substrate, and measured at 450 nm. The detection limit was 2.5 pg/mL.

*Analysis of plasma pTau181*: Blood was collected in EDTA vials, centrifuged (3,000 × *g* 5 min), and plasma-stored at minus 80°C until use. Plasma pT181 was analyzed using a novel Lumipulse assay (see https://www.fujirebio.com/en/products-solutions/lumipulse-g-ptau-181-plasma).

### 2.4 Statistical analysis

Sample size calculation for independent samples with a power of 80% and *α* = 0.05 revealed a minimal sample size of 12 per group. A proposed dropout rate of 25% increased the number per group to 15. To increase the credibility of data, the sample size was increased to 20 per group, resulting in a total of 80 probands. Data were analyzed for normality of distribution with the Shapiro–Wilk test, not resulting in a normal distribution. Statistical analysis was performed by using one-way ANOVA with a subsequent Fisher least significant difference (LSD) or a Dunnett *post-hoc* test. *p*-values <0.05 were considered significant. Data analysis was performed using SPSS software for Windows, Version 29.0.0.0 (SPSS Inc., Chicago, IL, USA). All evaluations were done on a blinded basis.

## 3 Results

### 3.1 Characterization of the study population and saliva samples

From a total of 82 participants, five saliva samples (four from edentulous subjects and one from the mild periodontitis group) were not suitable for analysis due to high viscosity or little quantity, so they were excluded. Thus, 77 individuals (42 females and 35 males; all Caucasians) were included in the study and allocated to the four groups. Salvia samples of 18 edentulous patients (10 females, 8 males), 20 subjects without advanced periodontal disease (13 females, 7 males), 19 mild periodontitis patients (8 females, 11 males), and 20 severe periodontitis (11 females, 9 males) subjects could be analyzed. The mean age was 66–71 years (range 61–92 years), with no significant difference between the groups. Also, salivary flow rates from 668 ± 109 to 849 ± 138 µL/min and salivary protein contents from 1.65 ± 0.15 to 2.06 ± 0.29 mg/mL did not differ statistically compared with controls without advanced periodontal disease (see Table 1).

**Table 1:** Epidemiology of patients and saliva characteristics.

Group	n/Male	Age (years)	Salivary flow (µL/min)	Protein (mg/mL)
Without periodontal disease	20/7	66.1 ± 1.5	769 ± 110	2.01 ± 0.44
Mild periodontitis	19/11	69.5 ± 1.9	849 ± 138	1.65 ± 0.15
Severe periodontitis	20/9	66.2 ± 1.0	636 ± 80	1.82 ± 0.30
Edentulous	18/8	71.8 ± 1.9	668 ± 109	2.06 ± 0.29

Values are given as mean ± SEM; n gives the number of analyzed patients. Statistical analysis was performed by using one-Way ANOVA with a subsequent Dunnett *post-hoc* test with a significance ≤0.05; n gives the number of samples. Statistical analysis was done against the group without advanced periodontal disease. Note that neither age nor salivary flow or protein content is significantly different from controls without advanced periodontal disease.

### 3.2 AD biomarkers in saliva

Salivary levels of all four AD biomarkers were corrected for 1 mg/mL total protein to exclude variations in salivary flow. The salivary levels of Aβ(40) and Aβ(42) and pTau181 were approximately 35–40 pg/mg total protein in the control group, while total tau levels were markedly higher (1,500 pg/mg total protein) (Table 2). There were no significant differences regarding salivary Aβ(40) and Aβ(42) and Tau between test groups and controls without advanced periodontal disease. In edentulous patients, the concentration of salivary pTau181 was highly significantly increased (81 ± 18, *p* = 0.0035), compared with controls without advanced periodontal disease (39 ± 5). There was a tendency (*p* = 0.050; Student’s t-test) that pTau181 was higher in males in the edentulous group: male = 91 ± 36 (*n* = 8) and female = 73 ± 18 (*n* = 10).

**Table 2:** Salivary Aβ(40), Aβ(42), Tau, and phospho-Tau-181 (pTau181) levels.

Group	*n*	Aβ(42) (pg/mg)	Aβ(40) (pg/mg)	Tau (pg/mg)	pTau181 (pg/mg)
No periodontal disease	20	35 ± 6	35 ± 6	1,456 ± 300	39 ± 5
Mild periodontitis	19	40 ± 9	45 ± 13	1,562 ± 240	37 ± 6
Severe periodontitis	20	32 ± 7	28 ± 6	1,136 ± 194	34 ± 4
Edentulous	18	31 ± 7	31 ± 10	1,234 ± 180	**81** **±** **18** ***p*** **=** **0.0035**

Values are given as mean ± SEM; n gives the number of analyzed patients. Statistical analysis was performed by using one-Way ANOVA with a subsequent Fisher LSD *post-hoc* test with a significance ≤0.05; n gives the number of samples. Statistical analysis was done against the group without advanced periodontal disease.

Bold letters indicate statistically significant values.

### 3.3 Blood plasma pTau181 levels

Plasma levels of pTau181 were very low and approximately 1.2 pg/ml in controls (Table 3). There were no significant differences regarding pTau181 between test groups and controls without advanced periodontal disease. In edentulous patients, the concentration of plasma pTau181 was significantly increased (2.1 ± 0.4, *p* = 0.0015), compared with controls without advanced periodontal disease (1.2 ± 0.07) (see Table 3).

**Table 3:** Plasma pTau181.

Group	*N*	pTau181 (pg/ml)
Without periodontal disease	10	1.2 ± 0.07 vs.
Mild periodontitis	9	1.3 ± 0.2 ns
Severe periodontitis	11	1.5 ± 0.1 ns
Edentulous	8	**2.1** **±** **0.4** ***p*** **=** **0.0015**

Values are given as mean ± SEM; n gives the number of analyzed patients. Statistical analysis was performed by using one-Way ANOVA with a subsequent Fisher LSD *post-hoc* test with a significance ≤0.05; n gives the number of samples. Statistical analysis was done against the group without advanced periodontal disease (vs.).

Bold letters indicate statistically significant values.

### 3.4 Cognitive assessment

Group comparison of neuropsychological measures at baseline revealed significant differences in verbal memory (learning, delayed recall), figural memory, and psychomotor speed (Trail Making test A) with lowest z-scores in edentulous patients. Significantly lower mean z-scores in edentulous patients were consistent with mild cognitive impairment but did not reach the threshold for major cognitive impairment. Word list learning was significantly decreased in edentulous patients compared with mild periodontitis (*p* ≤ 0.01). Word list delayed recall was significantly lower in edentulous patients compared with all groups (*p* ≤ 0.01). Constructional praxis savings was significantly decreased in the edentulous group compared with the two periodontitis groups (*p* ≤ 0.01). Trail A was significantly reduced in the edentulous group compared with the mild periodontitis group (*p* ≤ 0.05) and controls or the severe periodontitis group (*p* ≤ 0.01) (Table 4).

**Table 4:** Neuropsychological measurements.

*N*	Without peri	Mild peri	Severe peri	Edentulous	Test^b^	*p*-value
	11	9	11	8		
Age (years)	66 ± 9	68 ± 7	68 ± 5	69 ± 4	*H* = 2.8	0.408
Education (years)	13 ± 3	11 ± 5	12 ± 4	11 ± 3	*H* = 3.1	0.372
Female (%)	72.7	44.4	63.6	50.0	*χ*^2^ = 2.0	0.570
pTau 181 (pg/mL)	1.2 ± 0.3	1.3 ± 0.4	1.5 ± 0.4	2.1 ± 1.0	*H* = 5.4	0.139
MMSE (total score)	28.9 ± 1.1	28.4 ± 1.3	28.0 ± 0.8	28.7 ± 1.0	*H* = 4.5	0.206
Word list learning^a^	0.4 ± 1.0	1.2 ± 1.0	0.4 ± 1.1	−0.8 ± 1.3	*H* = 10.3	**0**.**016**
Word list delayed recall^a^	0.4 ± 0.8	0.6 ± 0.8	0.7 ± 1.0	−0.8 ± 1.4 **	*H* = 8.4	**0**.**038**
World list delayed recall savings^a^	0.4 ± 0.5	0.2 ± 0.7	0.4 ± 0.7	−0.6 ± 1.6	*H* = 5.0	0.165
Word list discriminability^a^	0.3 ± 0.5	0.3 ± 0.7	0.4 ± 0.7	−0.04 ± 1.3	*H* = 1.1	0.760
Constructional praxis^a^	−0.1 ± 1.1	−1.0 ± 1.1	−0.2 ± 1.2	−0.6 ± 1.2	*H* = 3.9	0.272
Constructional praxis delayed recall^a^	0.5 ± 1.1	−0.1 ± 1.4	−0.6 ± 1.5	−1.2 ± 1.1	*H* = 7.6	0.054
Constructional praxis savings^a^	0.7 ± 1.3	0.6 ± 1.6	−0.4 ± 1.1	−0.9 ± 1.1	*H* = 8.6	**0**.**034**
BNT^a^	0.5 ± 1.2	0.2 ± 0.9	0.4 ± 0.9	−0.03 ± 1.4	*H* = 2.6	0.455
Verbal fluency animals^a^	0.4 ± 1.1	0.5 ± 1.4	0.3 ± 1.0	−0.3 ± 0.6	*H* = 3.9	0.267
Verbal fluency s-words^a^	0.01 ± 0.10	0.3 ± 0.9	−0.1 ± 0.8	−0.6 ± 0.8	*H* = 3.4	0.322
Trail A^a^	0.5 ± 1.1	0.03 ± 1.2	0.6 ± 1.2	−1.2 ± 0.9 **	*H* = 11.9	**0**.**008**
Trail B^a^	0.7 ± 1.0	−0.06 ± 1.3	−0.01 ± 1.5	−0.3 ± 1.3	*H* = 2.4	0.488
CLOX (total score)	1.1 ± 0.4	1.7 ± 0.9	1.3 ± 0.9	2.2 ± 2.0	*H* = 4.2	0.240
GDS- 30 items (total score)	5.9 ± 5.3	3.0 ± 4.4	4.7 ± 2.7	6.5 ± 4.7	*H* = 4.5	0.208

Bold letters indicate statistically significant values.

BNT, Boston Naming Test; MMSE, Mini Mental State Examination; GDS, Geriatric Depression Scale; CLOX, clock drawing test; Co, Control; Peri, periodontitis.

^a^ z-score, BNT, values are mean ± SD; statistical analysis vs. HC; Df = 3 in all variables measured.

^b^ The Kruskal–Wallis test was used for metric and Chi-square test for nominal variables, **p < 0.01. Word list learning was significantly decreased in edentulous patients compared with those with mild periodontitis (**p ≤ 0.01). Word list delayed recall was significantly lower in edentulous patients compared with all groups (**p ≤ 0.01). Constructional praxis savings was significantly decreased in the edentulous group compared with the two periodontitis groups (**p ≤ 0.01). Trail A was significantly reduced in the edentulous group compared with those with mild periodontitis (*p ≤ 0.05) and controls or those with severe periodontitis (**p ≤ 0.01).

### 3.5 Characterization of saliva

**Figure 1:**
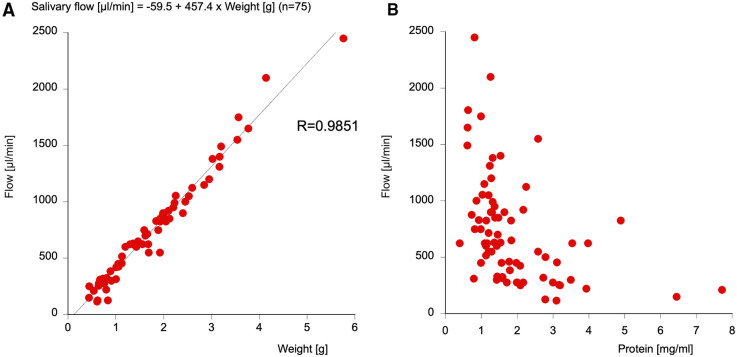
The correlation between the weight of the saliva and the salivary flow (**A**) and the correlation between total protein and salivary flow (**B**). Note that the salivary flow can be easily measured from the salivary weight using a formula with an R-value of 0.9851.

A directly proportional correlation between weight of saliva and salivary flow could be detected with a correlation coefficient of *r* = 0.99. Thus, with a formula [salivary flow (µL/min) = −59.5 + 457.4 × weight (g)], salivary flow can be directly calculated from the salivary weight. Total salivary protein content and salivary flow show an inversely proportional tendency, with high salivary flow correlating with little protein and low salivary flow correlating with high protein (see Figure 1).

### 3.6 Transferrin, cortisol, and interleukin-6

**Figure 2:**
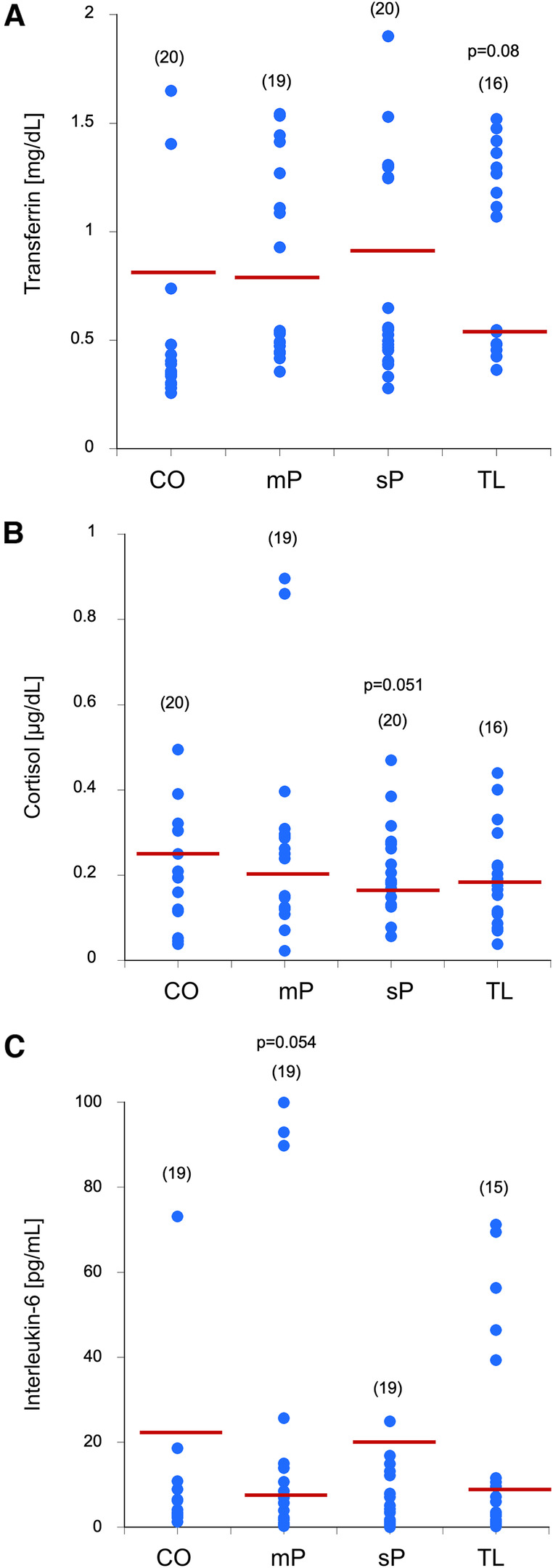
Blood contamination using transferrin (**A**), cortisol (**B**), and interleukin-6 (**C**) levels were measured in the saliva samples of controls without advanced periodontal disease (CO), mild peridontitis (mP), severe peridodontitis (sP), or edentulous patients (TL). Single values are given as dots, and the values in parenthesis give the number of patients. The red line indicates the mean value. Statistical analysis was performed by using one-Way ANOVA with a subsequent Fisher LSD *post-hoc* test with a significance p ≤ 0.05; Statistical analysis was done against the group without advanced periodontal disease. *p*-values close to significance are given in the graph.

To measure blood contamination, salivary transferrin was measured, and controls had approximately 0.8 mg/dL transferrin. No changes were seen between the groups, although there was a clear tendency (*p* = 0.05) for decreased transferrin in the edentulous group (Figure 2).

To see any circadian changes, salivary cortisol was measured and the controls had approximately 0.3 µg/dL cortisol. No changes were seen between the groups, although there was a tendency (*p* = 0.051) for decreased salivary cortisol in the severe periodontitis group (Figure 2).

To measure inflammation-related effects, salivary interleukin-6 levels were measured and controls had approximately 20 pg/mL IL-6. There was a relatively high variance in the control group, but no difference between the groups, but a tendency (*p* = 0.054) for decreased salivary IL-6 in the mild periodontitis group (Figure 2).

### 3.7 Detailed characterization of edentulous patients

Supplementary Table 1 shows the details of edentulous patients with regard to sex, age, loss of teeth, medications, and comorbidity.

## 4 Discussion

In the present study, we aimed to measure Alzheimer biomarkers in the saliva of patients with mild and severe periodontitis compared with edentulous patients. While Aβ(40) and Aβ(42) as well as total tau were not altered, pTau181 was significantly enhanced in the edentulous patient group.

### 4.1 Characterization of patients

With 42 out of 77 probands, there were slightly more females participating in this study than males, but there was no statistical difference between the groups regarding gender. Because of one proband with 92 years of age, the age range was high, but most subjects were between 60 and 80 years old, and the mean age of the groups was comparable. The results of this study may not be valid for all ethnicities, as all our probands were Caucasian. Strict inclusion and exclusion criteria allowed us to avoid the bias of known influencing factors, but still it is difficult to find a homogeneous group of people, as with advanced age, many different comorbidities may occur. In case of strongly deviating results, in addition to CPITN tooth mobility, furcation defect and a radiological assessment of bone loss were examined, so that complete periodontal staging and grading were possible.

### 4.2 Collection of saliva

In a previous recent study (22), it was found that small peptides (such as Aβ) were not recovered from the cotton of Salivettes®, which would not only impede the finding of our primary outcome measures but also falsify total protein content. Thus, we chose the direct spitting method into a cottonless Falcon tube for exactly 2 min for the collection of unstimulated whole saliva. As this represents an easy, exact, and reproducible sampling procedure, further studies should follow this protocol for comparable results. One significant finding is the strong correlation (R = 0.9851) of salivary weight and flow, so that in future studies, salivary flow can be simply calculated with the following formula: salivary flow (µL/min) = −59.5 + 457.4 × weight (g). It must be mentioned that strongly viscid saliva was not appropriate for processing for analysis. Also in some cases, the volume of the saliva sample was too low.

### 4.3 Blood contamination tested with transferrin

Because of higher levels of biomarkers in blood compared with those in saliva, we had to make sure that our results really represent the levels of biomarkers in saliva. Therefore, we used transferrin, the most reliable and valid marker for blood contamination in saliva (57), which showed no significant difference between control and test groups. This was important for us to show, as we can exclude the fact that the altered biomarkers in saliva do not come from the blood. It was interesting to note that edentulous patients even showed a tendency for reduced blood contamination, which could be due to the lack of dental micro-bleedings.

### 4.4 Diurnal variations tested with cortisol

Individuals with AD have basal cortisol elevations, but there is no evidence on changes in circadian cortisol levels (58). For these reasons, samples were taken between 9:00 and 14:00. No differences in cortisol levels between the control group and the edentulous or mild periodontitis group were found. There was a trend in the severe periodontitis group showing decreased salivary cortisol. A systematic review and meta-analysis (59) describes that aggressive periodontitis is associated with higher salivary cortisol levels compared with controls without advanced periodontal disease or patients with chronic periodontitis. Although the new classification scheme for periodontal diseases (60) does not distinguish those two types of periodontitis any more, in this case, the former classification seems reasonable. The participants showing higher cortisol levels were almost 20 years younger than patients with chronic periodontitis (mean 36.3 vs. 53.6 years). Considering the mid-thirties as the most stressful phase of life with multiple professional and private burdens, elevated cortisol levels are comprehensible. So, the reason that our probands with severe periodontitis showed tendentially lower cortisol levels may be that most of them were retired with a mean age of 66.2 years. In contrast, in light of the fact that in our study there was no age difference between the groups, the reduced cortisol levels in the severe periodontitis group may go hand in hand with hyperergic immune response and therefore worsen the proinflammatory state of periodontitis.

### 4.5 Inflammatory processes measured with interleukin-6

IL-6 is a ubiquitously expressed proinflammatory cytokine via trans-signaling and plays in important role in bone homeostasis (61). In a recent publication, it was described that salivary IL-6 levels are significantly higher in periodontitis stage III/IV compared with those in stage I/II or controls without advanced periodontal disease (62). Concerning mild and severe periodontitis, our results agree with the latest evidence, whereas we found contrary results when comparing periodontitis patients with controls without advanced periodontal disease. This may be due to the broad variance of IL-6 levels within our control group.

### 4.6 Beta-amyloid, tau, and pTau181

AD is the result of a very complex neurodegenerative process and is associated with the accumulation of Aβ in senile plaques and hyperphosphorylation of tau proteins (3). Based on a recent meta-analysis, significantly increased levels of Aβ(42) and pTau181 levels can be found in the saliva of AD patients (63). In our study, no changes were seen for Aβ in all groups, pointing that neither the toxic brain-derived Aβ(42) nor the blood-derived Aβ(40) may be secreted into the saliva. This further supports that the saliva was not bloody, as Aβ(40) is found in very high levels (10 ng/ml) in the blood, possibly playing a role in blood clotting, as it is released from platelets. In contrast, the levels of blood Aβ(42) are markedly lower (30–40 pg/ml). Tau levels in saliva are very high and this is in agreement with our previous study (22). Our data also show that tau is not affected in the saliva of periodontitis patients and also not in edentulous patients. The levels of pTau181 in saliva are very low as measured by Lumipulse. Here, we must consider that the Lumipulse cartridges and assay are validated for cerebrospinal fluid and not for saliva. This indeed may reflect an origin of failure to measure these four biomarkers. In the present study, we showed that pTau181 was significantly enhanced in the edentulous group compared with controls without advanced periodontal disease. These findings are novel and show for the first time that salivary levels of pTau181 are enhanced in edentulous patients. It is also interesting to note that out of the 18 edentulous patients, only 9 had very high levels (>35 pg/mg), while 9 had levels comparable to the controls (approximately 35 pg/mg). In this regard, several edentulous patients had extremely high levels of salivary pTau181, up to 164 pg/mg, and 5/18 had levels above 100 pg/mg. This is interesting to note, and more care must be taken to consider why and from where these high pTau181 levels come.

### 4.7 Salivary pTau181—from where does it come?

The important question, how periodontopathogens and their metabolites can reach and harm the brain, has recently been reviewed by us (23). The complex, bidirectional connections between saliva and the human brain via the oral-gut-brain axis can be summarized as follows: (1) the neural pathway with direct anatomic routes along several cranial nerves, (2) the intranasal pathway (64), (3) the lymphatic pathway, (4) the sublingual route, and (5) the transport of bacteria and inflammatory cytokines to the brain via the peripheral bloodstream. In addition, (6) the oral-gut-brain axis via the vagal nerve (65) must be considered. Saliva was carefully implemented by collecting unstimulated saliva after at least 2 h free from mechanical influences. This ensures the collection of non-diluted saliva, free from contamination by mechanically desquamated cells. In particular, in edentulous patients, contamination from peripheral circulation can also be ruled out, since in the absence of a periodontium, there is no sulcus fluid, which would otherwise represent a net filtrate of blood. So, the major question is, from where does tau come, a mechanism that we cannot fully explain in this study.

Tau is a neuronal protein and released in the brain after neurodegeneration and secreted into the cerebrospinal fluid, where its serves as a biomarker of cell death. Tau and pTau181 are also found in human plasma, reflecting the stage of AD progression. In the present study, we find high tau levels and altered pTau181 levels in the saliva of edentulous patients. The main question is, from where does the high pTau181 come? Although we cannot show evidence for it, we suggest the following source of pTau181. (1) Our data show that saliva is not contaminated from blood as there is no transferrin and Aβ(40) enhanced in saliva, so salivary pTau181 will not come from the blood. (2) It is possible that pTau181 is secreted from the salivary glands and lymph nodes and from there secreted into the saliva possibly also via the sublingual pathway. (3) It is possible that the degeneration of the neural tooth innervation in the edentulous patient is lost or damaged and subsequently reacts with an enhanced neuronal pTau181 expression. (4) Most likely, however, we think that tau/pTau181 comes from the oral buccal epithelium and/or the olfactory epithelium. Zuev et al. (66) suggested that tau in buccal epithelium is promising for the investigation of AD. Hattori et al. (67) reported that oral epithelia contain two tau isoforms, a small (full length) 65 kDa form and a large 110 kDa (pre) form and high tau in oral mucosal epithelium correlated with AD progression. Next, paired helical filaments (PHF) tau has been demonstrated in the olfactory epithelium of AD patients (68), especially in dystrophic olfactory neurons (69). Thus, our data are fully in line with this and may suggest that pTau181 comes from the buccal and/or the olfactory epithelium in edentulous patients.

### 4.8 Is salivary pTau181 the first sign of Alzheimer’s disease?

The central question in this study is why is the AD biomarker pTau181 statistically significant and elevated in the saliva of edentulous probands and are these results clinically relevant? A study including a total of 597 probands who have been followed up to 32 years showed that tooth loss, no matter whether the reason is caries or periodontal disease, is a predictor for cognitive decline in older men (37). A hypothesis could be generated that edentulous is the terminal stage of periodontitis, and despite proper extraction, periodontopathogens may remain in the bone and continue influencing peripheral diseases. Although their natural environment is the periodontium containing all its different tissues, the jawbone could be an alternative habitat for the typical anaerobic bacteria of the red cluster, which are characteristic of the development of periodontitis and associated with AD (43, 76). Another potential reason may be the reduced masticatory function of edentulous subjects—even when wearing complete dentures—resulting in a leaky gut, which is associated with a leaky blood–brain barrier (70) and thus promotes neuroinflammation. A meta-analysis revealed that patients suffering from AD have an increased risk of dental loss and edentulous condition compared with control groups (71). This confirms the bidirectional relationship between AD and edentulous patients.

Out of the included 18 edentulous patients, only 45% were willing to undergo a neuropsychological examination or attended the scheduled appointment. A total of 25% had cognitive deficits, whereby patients with very high phospho-tau181 values in particular did not want a neuropsychological examination. It can be assumed that especially these patients have a higher degree of cognitive deficits but refuse neuropsychological testing due to the frequent occurrence of anosognosia in the early stages of AD. The cause of tooth loss in our probands is described in Supplementary Table 1 and it includes periodontitis, caries, and trauma. There is no evidence that caries is an etiological factor for cognitive decline or elevated pTau181 levels; however, individuals with dementia often exhibit poor oral health, leading to a higher incidence of caries. Dental trauma as a cause of tooth loss itself cannot be considered a risk factor for AD symptoms or biomarkers, but the trauma may be associated with a potential traumatic brain injury, which could lead to a leaky blood–brain barrier and promote cerebral inflammation.

Definitely, these findings are of clinical relevance. As dentists monitor their patients for decades, they know each person very well, so that when changes in behavior or decline in oral hygiene occur, they realize it very quickly. The diminution of self-care and disability to follow simple instructions (as in dental prophylaxis) are important signs of mental impairment, and therefore it may be the dentist who detects a neurodegenerative process and initiates an examination at a professional unit for AD. Conversely, when a neurologist or a psychiatrist detects a very young person suffering from mild cognitive impairment (MCI) or AD, or attends on a person with rapid progression of disease, a dentist should be contacted for oral examination, and if applicable periodontal therapy, for reducing the risk of rapid destruction. In addition, for verifying the diagnosis and monitoring progress and therapy, periodic saliva samples should be taken and this should be considered a routine diagnostic exercise. This way of gathering human fluid for diagnosis is reasonable for every person, independent of the stage of disease, medication, or general health status and is neither painful nor has the risk of serious complications as the present standard method. This interdisciplinary collaboration offers a great opportunity to detect and treat people with mental constraints sooner and better, so that the intervention can happen very early in the process of degradation and therapy can rapidly react on any unwished changes as detected clinically or in the biomarker profile in saliva.

### 4.9 Limitations and outlook of the study

Definitely this study has limitations: (1) One marked limitation of the study is the limited number of probands and this study could be termed a “pilot study,” although we did a power analysis indicating that *n* = 12 should be appropriate. Thus, the broad distribution of different parameters in the control group represents a difficulty in our trial and some markers (especially cortisol, transferrin, or IL-6) could become statistically significant with a higher *n*-number. (2) The high variance may be due to different comorbidities and bears the question, “What is a good ‘healthy’ control without advanced periodontal disease.” Before checking the inclusion criteria, we had a checklist that contained medical history, medication, dental history and status, different habits, diseases, and conditions or operations, which could possibly further influence the measured biomarkers or periodontal and cognitive status. This information was important for us, because in case of extreme deviations, we can discuss the influence of different diseases on our topic of investigation. But still, within this study, it was not possible to obtain an overview of all comorbidities that could influence the complex interaction of periodontal status and AD; therefore, further studies are needed. (3) It was important that the average age of each group was quite similar, so that the results were comparable. But for further trials, it may be meaningful to have a strict age limitation from 60 to 80 years, or with a larger number of patients even to further segment each group in age with 5-year steps. (4) In our edentulous group, the reasons for tooth loss of the probands were not only periodontitis, but also caries or trauma, and it is not really possible to say from where pTau181 really comes. (5) In the present study, we focused on pTau181 because this assay has been previously used for saliva in our hands (22). It will be interesting to also measure other isoforms of pTau181, such as pTau217 with Lumipulse, but also more of the 40 phosphorylation sites of Tau would be of interest, which will be the aim of future studies. As the assays are validated for CSF and plasma only, future detailed studies must also test matrix effects and cross-reactivity in saliva samples. Although not proven, we do not think that the saliva matrix influences the results. (6) Definitely, future studies must also include other salivary specific markers, such as C-reactive protein or tumor necrosis factor-α or immunoglobulin-A or alpha-amylase. In this context, nanotechnology (72, 73) may help identify new associative markers in saliva. Another technical issue is that the total protein content may differ by disease state and may potentially bias the normalization, although it is unlikely. This should be verified in future experiments.

## 5 Conclusion

Matsumoto et al. (74) show for the first time that tooth loss may enhance AD tau pathology, promoting the spread of tau in the brain. Our data are fully in line with their findings and show for the first time that pTau181 is enhanced in the saliva and plasma of edentulous patients. This may represent a potential associative marker for the early detection of AD in toothless subjects, which could reliably support the early diagnosis of this neurodegenerative disease. Further, this is of clinical relevance as dentists could be the first people to discover early pathological changes in dental formation, which could lead to AD, and a prevention of dental loss may counteract AD progression.

## Data Availability

The original contributions presented in the study are included in the article/Supplementary Material, and further inquiries can be directed to the corresponding author.
