# Supplementary material for: Phospho-tau 181 is enhanced in saliva and plasma of edentulous patients: a first sign of dementia?
Source: Front Oral Health. 2025 Jul 30;6:1627681. doi: 10.3389/froh.2025.1627681 (PMC12343693; doi:10.3389/froh.2025.1627681)
Supplement: Supplementary file 1 [file Table1.pdf]

Supplementary Table 1: The edentulous patients

Nr.	Sex	Age [Y]	Saliva pTau181 [pg/mg]	Teeth lost since Y	Cause of tooth loss	History of periodontitis	Smoking (pcs/d)	Medication	Co-morbidity	Cognition
78	M	72	**303.6**	up 1999 low 2023	caries	yes	30	acetylsalicylic acid, amlodipine, valsartan	coronary hearth disease hypertension	intact
2	F	82	**163.8**	2022	periodontitis	yes	8	acetylsalicylic acid	stoma status post liver carcinoma	?
1	M	92	**159.3**	2019	periodontitis	yes	no	acetylsalicylic acid	coronary hearth disease	?
53	M	65	**147.0**	2023	caries	?	no			?
15	F	60	**132.7**	up 2002 low 2009	periodontitis	yes	15	clopidogrel insulin vitamin B complex	coronary hearth disease arteriosklerosis diabetes mellitus II cataract	?
4	M	70	**128.6**	up 1997 low 2008	periodontitis	yes	no	clopidogrel cortisone ciclosporin azathioprine	diabetes mellitus II status post kidney transplant	?
43	F	64	**77.1**	up 2003 low 2008	?	?	no	levothyroxine pantoprazole	hypothyroidism hypertension	**impaired**
68	F	76	**72.4**	up 2009 low 2022	caries periodontitis	yes	10	bisoprolol, duloxetine, ezetimibe, rosuvastatin, pantoprazole, lorazepam, alprazolam, trazodone, dimenhydrinate, magnesium	HI/RR? depression, anxiety disorder, vertigo hypercholesterolemia	?
45	F	76	**68.5**	1988	caries periodontitis	?	10	levothyroxine Carvedilol Theophyllin Tramadol	hypothyroidism COPD cardial insuffiency	intact
79	M	70	**41.7**	1994	?	?	no	acenocoumarol, nebivolol, levothyroxine, lisinopril, dutasteride , tamsulosin, atorvastatin	atrial fibrillation hypothyroidism hypertension benign prostate hyperplasia hypercholesterolemia	intact
63	M	64	**36.7**	2004	periodontitis	yes	no	levothyroxine candesartan Allopurinol	hypothyroidism hypertension	intact
59	F	70	25.8	2022	periodontitis	yes	no			**impaired**

77	M	71	24.7	2023	periodontitis	yes	no	metformin, sitagliptin, glimepiride, metoprolol, hydrochlorothiazide	diabetes mellitus II<br>hypertension	?
21	M	73	22.3	1992	caries	?	20		status post basal cell carcinoma	?
52	F	79	15.8	1998	periodontitis	yes	no	prednisolone	cataract	?
16	F	68	15.3	1992	caries	?	11	blood pressure medication	status post breast cancer<br>glaucoma<br>hypertension	intact
62	F	60	12.4	up<br>2004<br>low<br>2023	caries<br>periodontitis	yes	10-20	brotizolam	anxiety disorder<br>depression	?
64	M	69	12.0	?	?	?	no	mycophenolic acid, tacrolimus, amlodipine, ramipril, acetylsalicylic acid, calcium, vitamin D, fenoterol, ipratropium, fluticasone , vilanterol , umeclidinium	COPD II<br>status post liver transplant<br>coronary hearth disease<br>hypertension<br>status post aortic aneurysm<br>renal insufficiency	intact

This Table shows the details of the edentulous patients ranked by high salivary phospho-tau 181 (pTau181). Note that levels above 40 pg/mg are considered as increased as healthy controls have 39±5 pg/mg (n=20) pTau181 in saliva. Abbreviations: F, female; M, male; Y, years; pcs/d, peaces per day; up, upper jaw; low, lower jaw.
